# Genomic Characterization of the Cluster CZ4 Gordonia terrae Phage Oregano

**DOI:** 10.1128/mra.00679-22

**Published:** 2022-09-13

**Authors:** Hannah Lembree, Oyku Goktug, Dorian Royal, Alexander Russell, Annika Savage, Makayla Sisco, Maple Waltner, Veronica Chun, Melody N. Neely, Sally D. Molloy

**Affiliations:** a Molecular and Biomedical Sciences, University of Maine, Orono, Maine, USA; b The Honors College, University of Maine, Orono, Maine, USA; Queens College CUNY

## Abstract

Oregano is a novel cluster CZ4 bacteriophage isolated from the soil using the bacterial host Gordonia terrae. The Oregano genome is 47,575 bp long and encodes two tyrosine integrases and a toxin/antitoxin system. It shares an immunity repressor with both *Gordonia* and Mycobacterium phages that spans 7 clusters.

## ANNOUNCEMENT

**FIG 1:**
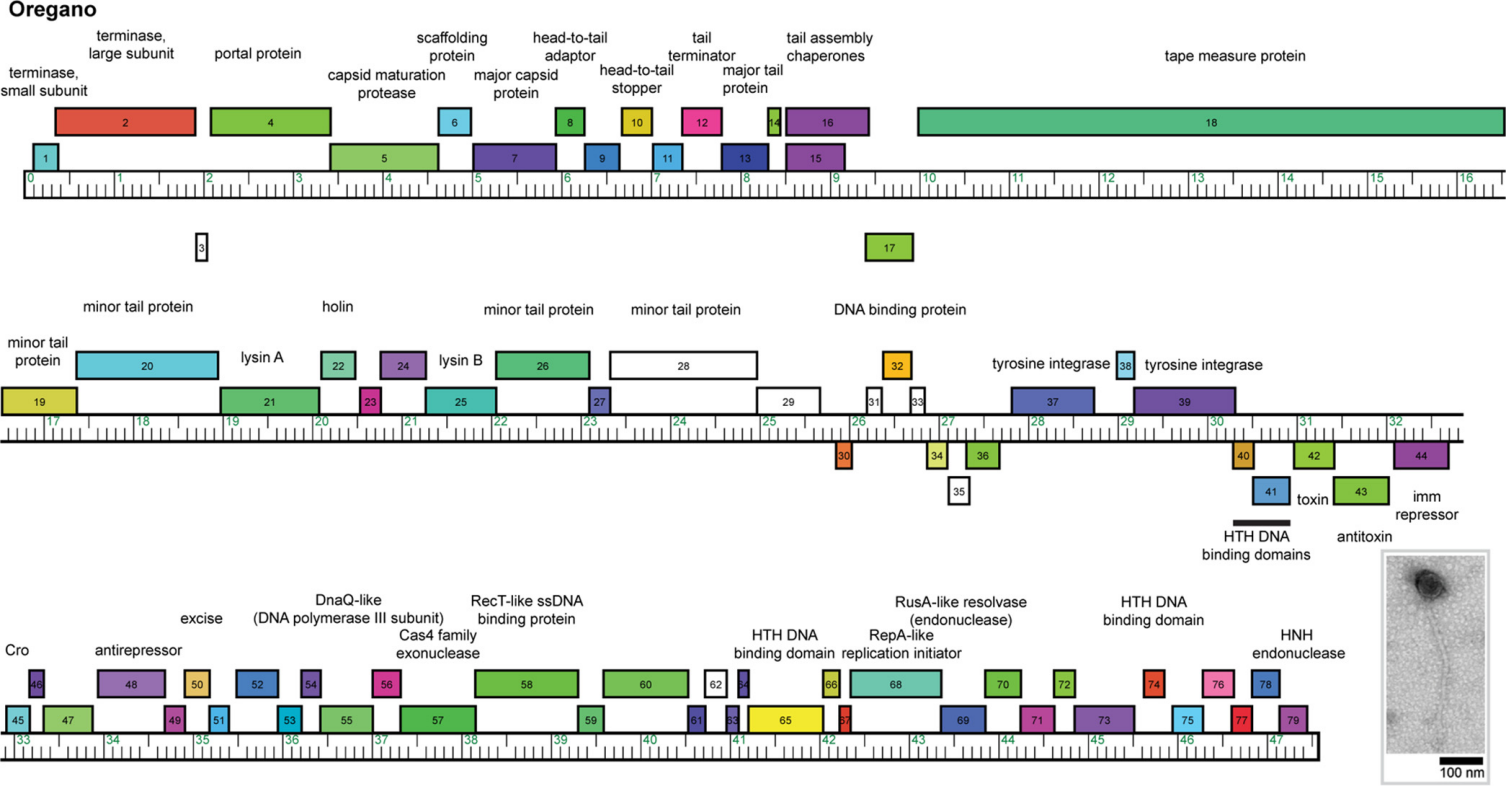
Genome map of *Gordonia* phage Oregano. The genome coordinates are represented by the ruler in units of kilobase pairs. The colored boxes above and below the ruler represent genes transcribed in the forward and reverse directions, respectively. Genes were assigned to a phamily using Phamerator (10) in the Actino_draft database, and different phamilies are indicated by different colors. Predicted functions are centered above and below forward- and reverse-transcribed genes, respectively. (Inset) Electron micrograph of Oregano. ssDNA, single-stranded DNA; HTH, helix-turn-helix; imm repressor, immunity repressor.

Actinobacteriophage are a diverse group of viruses that infect actinobacteria, a large group of Gram-positive bacteria that include both pathogenic and environmental bacteria relevant to human health (1, 2). By studying actinobacteriophage, we increase our understanding of the evolution and diversity of phage and their bacterial hosts (3–5). Bacteriophage Oregano was isolated from soil collected in Orono, ME (44.915628 N, 68.69072 W), using the actinobacterial host Gordonia terrae 3612. Soil extracts were prepared in peptone-yeast extract-calcium (PYCa) medium and filtered using an 0.22-μM filter. The filtrate was inoculated with *G. terrae* and incubated at 30°C for 2 days before being filtered, diluted, and plated in soft agar containing *G. terrae* onto PYCa agar. Oregano produced turbid plaques 1.0 mm in diameter after 2 days of incubation at 30°C. After five rounds of plaque purification using standard methods, the particle morphology of Oregano was determined by negative stained transmission electron microscopy (Fig. 1) (6). Oregano has a *Siphoviridae* morphology with a long, flexible, noncontractile tail 358.8 ± 4.4 nm (mean ± standard error [SE]) long and an icosahedral head 55.8 ± 0.5 nm (mean ± SE) in diameter (*n* = 4).

DNA was extracted from a high-titer lysate by phenol-chloroform extraction (7). DNA was prepared for sequencing using the Kapa Plus DNA library kit (Roche, South San Francisco, CA) and sequenced on an Illumina HiSeq platform. This yielded 500,000 paired-end 250-bp reads. Newbler v2.9 and Consed v29 were used to assemble the sequence and check it for completeness, respectively, yielding a 47,575-bp genome with 66.4% G+C content (8). The genome ends are defined by single-stranded 11-bp 3′ extensions (TGCCAAGGGGA). Based on shared gene content of 35% or higher with sequences in the Phamerator Actino_Draft database, Oregano was assigned to subcluster CZ4 (2, 9, 10).

Auto-annotation of Oregano’s genome was performed using DNA Master v5.23.6 (http://cobamide2.bio.pitt.edu/) and PECAAN (https://blog.kbrinsgd.org/) using the embedded programs GLIMMER v3.02 and GeneMark v2.5 (11, 12). Translational starts were refined using BLAST and Starterator (http://phages.wustl.edu/starterator/) by identifying conserved starts that included the coding potential predicted using GeneMark (13). Putative gene functions were predicted using BLAST, TMHMM, HHpred, and the Phamerator Actino_Draft database (10, 14, 15). No tRNA genes were identified using Aragorn v1.2.38 and tRNAscan-SE (16, 17). The genome contains 79 protein coding genes, of which 48% were assigned a function. The left arm encodes forward-transcribed structural and assembly genes (gp1 to gp29) (Fig. 1). The right arm of the genome contains forward-transcribed genes (gp45 to gp79), including Cro (gp45), an antirepressor (gp48), excise (gp50), and a RecT single-stranded DNA binding protein (gp58).

Between the minor tail proteins and Cro, there is a group of forward- and reverse-transcribed genes (genes *30* to *44*) that are likely expressed during lysogeny (18). These include two tyrosine integrases (gp37 and gp39), several DNA binding proteins (gp32, gp40, and gp41), and an immunity repressor (gp44). Oregano shares an immunity repressor with 41 *Gordonia* and Mycobacterium phages across six clusters (AD, CY, CZ, DH, DN, and P). Gp42 and gp43 are a putative toxin/antitoxin (TA) system. Gp42 and gp43 have strong HHpred matches to a PilT N-terminal (PIN) domain and an M. tuberculosis VapB antitoxin (PDB accession no. 5AF3_A), respectively (19). The TA system is found in six other phage genomes in clusters CZ4 and CZ6.

### Data availability.

Oregano is available at GenBank under the accession no. ON456355 and the Sequence Read Archive (SRA) accession no. SRX14816099.

## References

[1] Miao V, Davies J. 2010. Actinobacteria: the good, the bad, and the ugly. Antonie Van Leeuwenhoek 98:143–150. doi:10.1007/s10482-010-9440-6. 20390355

[2] Pope WH, Mavrich TN, Garlena RA, Guerrero-Bustamante CA, Jacobs-Sera D, Montgomery MT, Russell DA, Warner MH, Hatfull GF, Science Education Alliance-Phage Hunters Advancing Genomics and Evolutionary Science (SEA-PHAGES). 2017. Bacteriophages of Gordonia spp. display a spectrum of diversity and genetic relationships. mBio 8:e01069-17. doi:10.1128/mBio.01069-17. 28811342 PMC5559632

[3] Hatfull GF. 2018. Mycobacteriophages. Microbiol Spectr 6:GPP3-0026-2018. doi:10.1128/microbiolspec.GPP3-0026-2018. PMC6282025 30291704

[4] Jacobs-Sera D, Abad LA, Alvey RM, Anders KR, Aull HG, Bhalla SS, Blumer LS, Bollivar DW, Bonilla JA, Butela KA, Coomans RJ, Cresawn SG, D'Elia T, Diaz A, Divens AM, Edgington NP, Frederick GD, Gainey MD, Garlena RA, Grant KW, Gurney SMR, Hendrickson HL, Hughes LE, Kenna MA, Klyczek KK, Kotturi H, Mavrich TN, McKinney AL, Merkhofer EC, Moberg Parker J, Molloy SD, Monti DL, Pape-Zambito DA, Pollenz RS, Pope WH, Reyna NS, Rinehart CA, Russell DA, Shaffer CD, Sivanathan V, Stoner TH, Stukey J, Sunnen CN, Tolsma SS, Tsourkas PK, Wallen JR, Ware VC, Warner MH, Washington JM, Westover KM, et al. 2020. Genomic diversity of bacteriophages infecting Microbacterium spp. PLoS One 15:e0234636. doi:10.1371/journal.pone.0234636. 32555720 PMC7302621

[5] Pope WH, Jacobs-Sera D, Russell DA, Peebles CL, Al-Atrache Z, Alcoser TA, Alexander LM, Alfano MB, Alford ST, Amy NE, Anderson MD, Anderson AG, Ang AA, Ares M, Jr, Barber AJ, Barker LP, Barrett JM, Barshop WD, Bauerle CM, Bayles IM, Belfield KL, Best AA, Borjon A, Jr, Bowman CA, Boyer CA, Bradley KW, Bradley VA, Broadway LN, Budwal K, Busby KN, Campbell IW, Campbell AM, Carey A, Caruso SM, Chew RD, Cockburn CL, Cohen LB, Corajod JM, Cresawn SG, Davis KR, Deng L, Denver DR, Dixon BR, Ekram S, Elgin SC, Engelsen AE, English BE, Erb ML, Estrada C, Filliger LZ, et al. 2011. Expanding the diversity of mycobacteriophages: insights into genome architecture and evolution. PLoS One 6:e16329. doi:10.1371/journal.pone.0016329. 21298013 PMC3029335

[6] Poxleitner M, Pope W, Jacobs-Sera D, Sivanathan V, Hatfull G. 2018. Phage discovery guide. Howard Hughes Medical Institute, Chevy Chase, MD.

[7] Sambrook J, Russell DW. 2006. Purification of nucleic acids by extraction with phenol: chloroform. CSH Protoc 2006:pdb.prot4455. doi:10.1101/pdb.prot4455. 22485786

[8] Gordon D, Green P. 2013. Consed: a graphical editor for next-generation sequencing. Bioinformatics 29:2936–2937. doi:10.1093/bioinformatics/btt515. 23995391 PMC3810858

[9] Russell DA, Hatfull GF. 2017. PhagesDB: the actinobacteriophage database. Bioinformatics 33:784–786. doi:10.1093/bioinformatics/btw711. 28365761 PMC5860397

[10] Cresawn SG, Bogel M, Day N, Jacobs-Sera D, Hendrix RW, Hatfull GF. 2011. Phamerator: a bioinformatic tool for comparative bacteriophage genomics. BMC Bioinformatics 12:395. doi:10.1186/1471-2105-12-395. 21991981 PMC3233612

[11] Besemer J, Borodovsky M. 2005. GeneMark: Web software for gene finding in prokaryotes, eukaryotes and viruses. Nucleic Acids Res 33:W451–W454. doi:10.1093/nar/gki487. 15980510 PMC1160247

[12] Delcher AL, Bratke KA, Powers EC, Salzberg SL. 2007. Identifying bacterial genes and endosymbiont DNA with Glimmer. Bioinformatics 23:673–679. doi:10.1093/bioinformatics/btm009. 17237039 PMC2387122

[13] Altschul SF, Madden TL, Schäffer AA, Zhang J, Zhang Z, Miller W, Lipman DJ. 1997. Gapped BLAST and PSI-BLAST: a new generation of protein database search programs. Nucleic Acids Res 25:3389–3402. doi:10.1093/nar/25.17.3389. 9254694 PMC146917

[14] Krogh A, Larsson B, Von Heijne G, Sonnhammer EL. 2001. Predicting transmembrane protein topology with a hidden Markov model: application to complete genomes. J Mol Biol 305:567–580. doi:10.1006/jmbi.2000.4315. 11152613

[15] Söding J, Biegert A, Lupas AN. 2005. The HHpred interactive server for protein homology detection and structure prediction. Nucleic Acids Res 33:W244–W248. doi:10.1093/nar/gki408. 15980461 PMC1160169

[16] Laslett D, Canback B. 2004. ARAGORN, a program to detect tRNA genes and tmRNA genes in nucleotide sequences. Nucleic Acids Res 32:11–16. doi:10.1093/nar/gkh152. 14704338 PMC373265

[17] Lowe TM, Eddy SR. 1997. tRNAscan-SE: a program for improved detection of transfer RNA genes in genomic sequence. Nucleic Acids Res 25:955–964. doi:10.1093/nar/25.5.955. 9023104 PMC146525

[18] Jacobs-Sera D, Marinelli LJ, Bowman C, Broussard GW, Guerrero Bustamante C, Boyle MM, Petrova ZO, Dedrick RM, Pope WH, Modlin RL, Hendrix RW, Hatfull GF, Science Education Alliance Phage Hunters Advancing Genomics and Evolutionary Science SEA-PHAGES Program. 2012. On the nature of mycobacteriophage diversity and host preference. Virology 434:187–201. doi:10.1016/j.virol.2012.09.026. 23084079 PMC3518647

[19] Arcus VL, McKenzie JL, Robson J, Cook GM. 2011. The PIN-domain ribonucleases and the prokaryotic VapBC toxin–antitoxin array. Protein Eng Des Sel 24:33–40. doi:10.1093/protein/gzq081. 21036780

